# A comparative study of SMILES-based compound similarity functions for drug-target interaction prediction

**DOI:** 10.1186/s12859-016-0977-x

**Published:** 2016-03-18

**Authors:** Hakime Öztürk, Elif Ozkirimli, Arzucan Özgür

**Affiliations:** Department of Computer Engineering, Bogazici University, Bebek, Istanbul, 34342 Turkey

**Keywords:** Chemoinformatics, SMILES, SMILES based drug similarity, Drug-target interaction prediction

## Abstract

**Background:**

Molecular structures can be represented as strings of special characters using SMILES. Since each molecule is represented as a string, the similarity between compounds can be computed using SMILES-based string similarity functions. Most previous studies on drug-target interaction prediction use 2D-based compound similarity kernels such as SIMCOMP. To the best of our knowledge, using SMILES-based similarity functions, which are computationally more efficient than the 2D-based kernels, has not been investigated for this task before.

**Results:**

In this study, we adapt and evaluate various SMILES-based similarity methods for drug-target interaction prediction. In addition, inspired by the vector space model of Information Retrieval we propose cosine similarity based SMILES kernels that make use of the Term Frequency (TF) and Term Frequency-Inverse Document Frequency (TF-IDF) weighting approaches. We also investigate generating composite kernels by combining our best SMILES-based similarity functions with the SIMCOMP kernel. With this study, we provided a comparison of 13 different ligand similarity functions, each of which utilizes the SMILES string of molecule representation. Additionally, TF and TF-IDF based cosine similarity kernels are proposed.

**Conclusion:**

The more efficient SMILES-based similarity functions performed similarly to the more complex 2D-based SIMCOMP kernel in terms of AUC-ROC scores. The TF-IDF based cosine similarity obtained a better AUC-PR score than the SIMCOMP kernel on the GPCR benchmark data set. The composite kernel of TF-IDF based cosine similarity and SIMCOMP achieved the best AUC-PR scores for all data sets.

**Electronic supplementary material:**

The online version of this article (doi:10.1186/s12859-016-0977-x) contains supplementary material, which is available to authorized users.

## Background

Identification of potential interactions between target proteins and drugs is a difficult task and computer scientists and medicinal chemists alike consider it a challenge before the whole drug discovery field. Efficient prediction of target-compound interactions using computational methods accelerates research efforts in this area. There have been two generally accepted approaches to drug discovery, ligand-based and structure-based or docking [1]. Ligand-based approaches are based on the known ligands of a protein and lack applicability when the target has no known ligands (orphan target), while structure-based approaches utilize the three dimensional structure of the target, if it is known [1]. Literature mining, where interacting genes and compounds are extracted from the related articles, can also be used [2]. Chemogenomics relates the chemical properties of ligands with the sequence properties of proteins, with the final goal of protein-ligand interaction prediction. The underlying assumption is that chemically similar compounds will bind to the same or similar proteins and that targets with similar binding sites should also bind to the same ligands [3]. In this work, we combine protein sequence similarity and ligand chemical similarity information for protein-drug interaction prediction using machine learning. Our main goal is to evaluate the performance of different ligand similarity functions that utilize their SMILES strings for this task.

Chemogenomics has three main components: (i) set of compounds (ii) set of targets (iii) reliable interaction information [4]. Recent studies have adopted chemogenomics approaches for predicting drug-target interactions [5–17] based on genomic similarity of proteins and/or chemical similarity of ligands using different computational models and classification algorithms. One of the first studies utilizing machine learning methods for ligand-based virtual screening tested different target kernels with Support Vector Machines (SVM). Three different protein data sets were examined to predict drugs even for targets with no known ligands using the similarity between proteins [6]. The same year, chemical compound similarity and protein sequence similarity were used to propose three different profile kernel methods by Yamanishi et al. for interaction prediction [5]. The database of drug-target interactions curated in this work is commonly used as a reference/benchmark data set [5]. Gaussian Interaction Profile (GIP) kernel, introduced by Laarhoven et al., was built on binary vectors named interaction profiles that are defined for each drug and protein in the data set [16]. The interaction profile of a drug indicates its interaction status (present (1)/ absent (0)) with every target in the data set. Similarly, the interaction profile of a target is a binary vector representing its interaction status with all the drugs in the data set. The model was later improved using the Weighted Nearest Neighbor algorithm (WNN-GIP), so that it was possible to make predictions for new drug compounds by predicting their interaction profiles [17].

The chemical similarity of ligands can be based on the 1D, 2D, and 3D representations of molecules [4]. The most commonly used descriptors are based on 2D and 3D representations of the compounds [18–22]. A detailed comparison of different chemical descriptors and 2D graph similarity kernels used in predicting protein-drug interactions was recently reported in [23]. A popular 1D representation for molecular structures is the Simplified Molecular Input Line Entry System (SMILES) that describes molecular structures in the form of strings [24, 25]. SMILES strings convey information about molecular structures by using symbols such as C, c, N, O for atoms and =, # for bonds (www.daylight.com/dayhtml/doc/theory/theory.smiles.html). The SMILES representation has been used to obtain molecular similarity for purposes such as toxicity prediction, virtual screening, and Quantitative structure-activity relationship (QSAR) modelling [26–30]. To the best of our knowledge, SMILES strings have not been previously used to represent similarities among compounds for the task of drug-target interaction prediction. Most previous studies on drug-target interaction prediction make use of the more complex 2D representations of the compounds such as SIMCOMP.

In this study, we seek to answer whether a SMILES representation based compound similarity method can perform as well as the widely used 2D representation based similarity method, SIMCOMP [31], in the drug-target interaction prediction task. The methods we discussed in this paper can be used in any drug-target interaction prediction algorithm that makes use of the compound similarity information. For evaluation purposes, we use one of the state-of-the-art algorithms for drug-target interaction prediction, namely the Weighted Nearest Neighbor-Gaussian Interaction Profile (WNN-GIP) model proposed in [17] and we use the benchmark data sets curated by Yamanishi et al.; GPCRs, enzymes, nuclear receptors, ion channels, and their interacting ligands [5]. We adapt and evaluate various string similarity functions, which are based on the SMILES representations of the ligands, for the task of drug-target interaction prediction. The string similarity methods that we utilize include edit distance [32], normalized longest common subsequence (NLCS), and a model that combines three different longest common subsequence (LCS) [33] algorithms, as well as SMILES specialized algorithms such as LINGO [26], SMILES fingerprint (SMIfp) [28], and SMILES-based substring kernel [27]. We also present two novel models that combine the LINGO representation with the term frequency (TF) weighting and term frequency-inverse document frequency (TF-IDF) weighting schemes adopted from the Information Retrieval domain. In addition, two composite kernels are constructed by combining the 2D-based similarity kernel SIMCOMP with TF-IDF based cosine similarity kernel and LINGOsim kernel. Our results show that SMILES-based similarity kernels perform close to the 2D-based similarity kernel, SIMCOMP, at a fraction of the computational time. The composite kernel comprising the SMILES-based TF-IDF cosine similarity kernel and 2D-based SIMCOMP kernel obtained the best performance in terms of AUC-PR scores for all data sets.

## Methods

In this section we first describe the data sets that we used for evaluation and the drug-target interaction prediction algorithm (i.e., Weighted Nearest Neighbor-Gaussian Interaction Profile (WNN-GIP) [17]), into which we integrated the SMILES-based compound similarity functions. Next, we present the SMILES-based compound similarity functions that we adapted and evaluated for the task of drug-target interaction prediction.

### Data sets

#### Drug-target interaction data

We used the benchmark drug-target interaction data sets for enzymes, ion channels, GPCRs, and nuclear receptors provided by Yamanishi et al. [5] for evaluation. The data sets are publicly available at http://web.kuicr.kyoto-u.ac.jp/supp/yoshi/drugtarget/. Yamanishi et al. retrieved the interaction information between target proteins and compounds from the KEGG BRITE [34], BRENDA [35], SuperTarget [36], and DrugBank [37] databases. The properties of the interaction data sets are provided in Table 1.

**Table 1:** Number of components included in the drug-target interaction data sets of Yamanishi et al. [5]

Dataset	Drugs	Targets	Interactions
Enzyme	445	664	2926
Ion Channels	210	204	1476
GPCR	223	95	635
Nuclear Receptor	54	26	90

#### Genomic data

Target proteins are retrieved from the KEGG GENES database [34], and the normalized version of the Smith-Waterman score is used to calculate the similarity between the amino-acid sequences of the target proteins [5].

#### Chemical data

The DRUG and COMPOUND sections of KEGG DRUG database [34] are used to obtain the chemical structures. SIMCOMP is used to construct the original compound similarity matrix [5]. SIMCOMP treats chemical molecules as graphs, then produces a score representing the similarity between two graphs [31, 38]. In order to calculate our own compound similarity matrices, we first downloaded the MOL files from KEGG DRUG for each of the compounds included in the data sets. Then, we used JChem 6.0.2 for.NET (ChemAxon, https://www.chemaxon.com/) to convert the MOL files into unique SMILES strings [25].

### Prediction algorithm

We used the WNN-GIP drug-target interaction prediction model [16, 17] in order to compare the different SMILES string based compound similarity methods.WNN-GIP requires a bipartite drug-target interaction network, which is represented as an adjacency matrix in which a cell is set to 1 if the drug and the target interact, and to 0 otherwise. Each row of the adjacency matrix corresponds to the interaction profile of a drug and each column corresponds to the interaction profile of a target. The interaction profiles of the drugs and targets, as well as the chemical similarity information of the compounds and the genomic similarity information of the proteins are provided as inputs to the WNN-GIP algorithm. First, the kernels for the drugs and targets are created from the chemical similarity and genomic similarity information, respectively by modifying their similarity matrices so that they become symmetric and positive definite (i.e., well-defined kernel functions). Then, the Gaussian kernel is used to construct a kernel from the interaction profiles, which is combined with the chemical kernel and the genomic kernel. Finally, the Kronecker product is used to merge the kernel for drugs and the kernel for targets into a kernel directly representing drug-target pairs. The Regularized Least Squares (RLS) algorithm is employed for the prediction of drug target interactions [16]. In the original study, WNN-GIP used the SIMCOMP method based on the 2D representation of the compounds to compute the compound chemical similarity scores. In this study, we investigated using the computationally more efficient 1D SMILES-based similarity functions to compute the compound similarity scores instead of SIMCOMP in the WNN-GIP algorithm.

### SMILES-based compound similarity functions

In this sub-section we provide the methods that we adopted and evaluated to measure the similarity of compounds using their SMILES string representations. The source code comprising the SMILES-based similarity algorithms discussed in this section is publicly available at: https://github.com/hkmztrk/SMILESbasedSimilarityKernels.

All SMILES strings are modified such that atoms represented with two characters such as ‘Cl’ and ‘Br’ are replaced with single characters. For illustration purposes, we use the imaginary SMILES strings *SMI*_1_: “OC(O) =O” and *SMI*_2_: “CCCCC(O) =C4” to demonstrate the SMILES-based similarity methods.

#### Edit distance

Edit distance is one of the most widely used measures to make comparisons between strings. Given two strings *S*_1_ and *S*_2_, the edit distance between them is defined as the number of minimum edit operations required to convert *S*_1_ into *S*_2_ [39]. There are three main operations allowed, namely insertion, deletion, and substitution [39]. For our samples, *edit*(*SMI*_1_,*SMI*_2_)=6. In order to convert *SMI*_1_ to *SMI*_2_, we have to perform four insertion operations (for inserting three characters “C” and for inserting one character “4”), and two substitution operations (for substituting the first and last “O” with “C”). Then, the similarity is calculated as,

$${} {\fontsize{8.9pt}{9.6pt}\begin{aligned} EditSim(SMI_{1},SMI_{2}) = 1 - \frac {edit(SMI_{1}, SMI_{2})} {MAX(length(SMI_{1}), length(SMI_{2}))} \end{aligned}} $$

#### Normalized longest common subsequence (NLCS)

The Longest Common Subsequence (LCS) algorithm finds the common subsequence with the maximum possible length of two strings [40]. The algorithm does not require the characters in the common subsequence to be consecutive. Normalized LCS modifies the algorithm such that the lengths of both strings are considered. Given two strings *S*_1_ and *S*_2_ the NLCS is [33],

$$NLCS(S_{1}, S_{2}) = \frac{length\left(LCS\left(S_{1}, S_{2}\right)\right)^{2}}{length\left(S_{1}\right) \times length\left(S_{2}\right)}$$

For our sample SMILES strings, the longest common subsequence is “C(O) =”. Therefore,

$$NLCS\left(SMI_{1}, SMI_{2}\right) = 0.32$$

#### Combination of LCS models (CLCS)

Islam and Inkpen proposed a method to measure semantic similarity of the texts by combining three algorithms each of which modifies the LCS algorithm in a different way [33]. We will refer to this method as CLCS throughout the paper. The first algorithm is Normalized LCS (NLCS), described in the previous section. The second algorithm is called Maximal Consecutive Longest Common Subsequence starting from the character 1, *MCLCS*_1_. It requires the common subsequences to be consecutive and to start from the first index of the shorter string. The last algorithm is named Maximal Consecutive Longest Common Subsequence starting from character n, *MCLCS*_*n*_ [33]. Similarly to *MCLCS*_1_, *MCLCS*_*n*_ requires the common subsequences to be successive. However unlike *MCLCS*_1_, the consecutive symbols don’t have to start from the first index in the *MCLCS*_*n*_, i.e, they can start from any position in the string. *MCLCS*_1_ and *MCLCS*_*n*_ are also normalized and named as *NMCLCS*_1_ and *NMCLCS*_*n*_, respectively. Given two strings *S*_1_ and *S*_2_, *NMCLCS*_1_ and *NMCLCS*_*n*_ are calculated as [33],

$$NMCLCS_{1}(S_{1}, S_{2}) = \frac{length\left(MCLCS_{1}\left(S_{1}, S_{2}\right)\right)^{2}}{length\left(S_{1}\right) \times length\left(S_{2}\right)}$$

$$NMCLCS_{n}(S_{1}, S_{2}) = \frac{length\left(MCLCS_{n}\left(S_{1}, S_{2}\right)\right)^{2}}{length(S_{1}) \times length(S_{2})}$$

In order to compute the similarity between *S*_1_ and *S*_2_, the weighted sum of these three algorithms is taken as follows [33]:

*K*(*S*_1_,*S*_2_)=*v*_1_×*w*_1_+*v*_2_×*w*_2_+*v*_3_×*w*_3_, where *w*_1_, *w*_2_, *w*_3_ are the weights and

$$\begin{array}{*{20}l} v_{1}& = NLCS\left(S_{1}, S_{2}\right)  \\ v_{2}& = NMCLCS_{1}\left(S_{1}, S_{2}\right)  \\ v_{3}& = NMCLCS_{n}\left(S_{1}, S_{2}\right)  \\ \end{array} $$

The original method gives each algorithm the same weight (*w*_1_=*w*_2_=*w*_3_=0.33)[33].

Let us demonstrate this model with our sample SMILES strings. For the first algorithm, *NLCS*(*SMI*_1_,*SMI*_2_)=0.32. *NMCLCS*_1_ requires the longest common subsequence to be consecutive and to start from the first index, which is equal to “O” and therefore, *NMCLCS*_1_(*SMI*_1_,*SMI*_2_)=0.012. For *NMCLCS*_*n*_, the longest common subsequence is “C(O) =”. *NMCLCS*_*n*_(*SMI*_1_,*SMI*_2_)=0.32. Finally, the similarity score becomes 0.218.

#### SMILES representation-based string kernel

SMILES representation-based string kernel is proposed as a compound similarity kernel and combined with SVM to predict in silico toxicity of the compounds in [27]. Given two SMILES texts *S*_1_ and *S*_2_, *θ*(*S*_1_) and *θ*(*S*_2_) respectively denote the frequencies of all the possible substrings with at least *q*=2 character length. The string similarity kernel is defined as the inner product of these frequencies [27].

$$K\left(S_{1}, S_{2}\right) = \langle \theta \left(S_{1}\right), \theta \left(S_{2}\right) \rangle $$

Consider our sample SMILES strings *SMI*_1_ and *SMI*_2_. The frequency of each *SMI*_1_ substring, {OC, C(, (O, …, OC(, C(O, …, OC(O, C(O), …, OC(O), C(O) =, (O) =O, OC(O) =, C(O) =O, OC(O) =O }, is 1. The frequency of the *SMI*_2_ substrings {C(, (O, O), …,CC(, C(O,…, CCCCC(O) =C, CCCC(O) =C4, CCCCC(O) =C4 } is also 1 except for the *SMI*_2_ substrings {CC, CCC, CCCC } that have frequencies of 4, 3, and 2, respectively. The shared substrings from these sets are, {C(, (O, O),) =, C(O, (O), O) =, C(O), (O) =, C(O) = }, all of which have a frequency of 1. Therefore, the inner product, *K*(*SMI*_1_,*SMI*_2_), is 10.

#### SMILES fingerprint (SMIfp)

SMILES Fingerprint (SMIfp) was introduced by Schwartz et al. to perform ligand-based virtual screening [28]. SMIfp is based on representing SMILES strings in a 34-dimensional space where each of the dimensions correspond to the frequency of a different symbol such as C, c, N, and # [28]. More than 32 million compounds in PubChem are analyzed in order to identify the most-frequent symbols in a SMILES string to form the best-representative scalar fingerprint and as a result, 34 relevant symbols are selected. Once SMILES strings are converted to scalar fingerprints, City Block Distance (CBD) [41] is used to measure similarities. Aside from CBD, we use Tanimoto coefficient to observe whether the distance metric affects the performance of the model.

CBD treats the common absence or low values of features as an indication of similarity, whereas Tanimoto does not [18]. On the other hand, unlike CBD, Tanimoto considers size normalization. For the chemical domain, Tanimoto is recommended to be used to measure the absolute similarity between two molecules, whereas CBD and Euclidian distance are useful to measure relative similarity, i.e. the relative similarities of two molecules to the some other molecule [42].

#### LINGO

LINGO refers to q-character substrings of a SMILES text [26]. LINGO representation of compounds has been used as input for Quantitative Structure-Property Relationships (QSPR) models as well as for calculation of intermolecular similarities. A SMILES string of length *n* can be represented with (*n*−(*q*−1)) q-length substrings (LINGOs). The original method requires SMILES strings to be canonical, and LINGO length is fixed as *q*=4. Before the LINGO creation process, all ring numbers in the SMILES string are set to ‘0’. Then, the LINGOsim function is used to calculate the similarity between two SMILES strings *S*_1_ and *S*_2_ with the Tanimoto coefficient based on their LINGO profiles [26],

$$LINGOsim = \frac { \sum_{i=1}^{m} 1 - \frac { \left| N_{S_{1},i} - N_{S_{2},i} \right|} { \left| N_{S_{1},i} + N_{S_{2},i} \right|} } {m}$$

where *m* is the total number of unique LINGOs created from *S*_1_ and *S*_2_, while $N_{S_{1},i}$ represents the frequency of LINGOs of type *i* in compound *S*_1_ and $N_{S_{2},i}$ represents the frequency of LINGOs of type *i* in compound *S*_2_.

Let us demonstrate how compound similarity is calculated using the LINGO model with our sample strings, *SMI*_1_ and *SMI*_2_. *SMI*_1_: “OC(O) =O” doesn’t require any modification, whereas for *SMI*_2_: “CCCCC(O) =C4” we have to set ring numbers to 0 and the string is converted into the following form, “CCCCC(O) =C0”. We then create LINGOs with the substring length of *q*=4.

Once we extract the LINGOs from the SMILES strings and retrieve their corresponding term frequencies (Table 2), we calculate LINGOsim. We have nine unique LINGOs two of which are shared by both SMILES strings.

$${} {\fontsize{8.4pt}{9.6pt}\begin{aligned} LINGOsim &= \frac { \sum_{i=1}^{9} 1 - \frac { \left| N_{SMI_{1},i} - N_{SMI_{2},i} \right|} { \left| N_{SMI_{1},i} + N_{SMI_{2},i} \right|} } {9} \\ &= \frac { \sum_{i=1}^{6}\!\! \left(\!1 \,-\, \frac { \left| 1 - 0 \right|} {\left| 1 + 0 \right|}\!\right)\! +\! \sum_{i=7}^{8}\! \left(\!1 - \frac { \left| 1 - 1 \right|} {\left| 1 + 1 \right|}\right)\! +\! \sum_{i=9}^{9} \!\left(\!1 - \frac { \left| 2 - 0 \right|} {\left| 2 + 0 \right|}\!\right)} {9} \\ &= \frac { 0 + 2 + 0} { 9} = 0.22 \end{aligned}} $$

**Table 2:** LINGOs with their corresponding frequencies in the sample SMILES strings *S* *M* *I*_1_ and *S* *M* *I*_2_

***S*** ***M*** ***I***_**1**_		***S*** ***M*** ***I***_**2**_	
LINGO	Freq	LINGO	Freq
OC(O	1	CCCC	2
C(O)	1	CCC(	1
(O) =	1	CC(O	1
O) =O	1	C(O)	1
		(O) =	1
		O) =C	1
		) =C0	1

LINGO length *q*=3,4,5 are tested in this work.

#### LINGO based TF cosine similarity

Term-Frequency (TF) based cosine similarity is the first of the weighting models that we adopted from the Information Retrieval domain. TF reflects the number of times a term occurs in a document [43]. Originally in TF weighting, a weight representing the frequency of a term is assigned to each term in the document. In this domain, we treat each SMILES string as a document and four character LINGOs, which are created from these strings, are denoted as terms. The TF weight of a LINGO *L* in SMILES string *S* is calculated as follows.

$${} \begin{aligned} TF&weight_{L, S} \\ &=\left\{\begin{array}{ll} \!\!1 +\! log_{10} (termfrequency_{L, S}), & \text{if}~termfrequency_{L, S} > 0\\ \!\!0, & \text{otherwise} \end{array}\right. \end{aligned} $$

In order to compute the similarity between two SMILES strings using this method, each string has to be converted into a feature vector *V*_*s*_. The dimensionality of *V*_*s*_ is equal to the number of unique terms (LINGOs) in the corpus (compound data set). Each feature contains the TF score of the corresponding term (LINGO) in the string (SMILES). The similarity of two SMILES strings *S*1 and *S*2 is determined according to the cosine angle between their vectors.

(1) $$ CosSim(S1, S2) = \frac { \sum_{i=1}^{m} V_{S1,i} V_{S2,i}} {\lVert V_{S1}\rVert \lVert V_{S2}\rVert }  $$

*V*_*S*1_ and *V*_*S*2_ are feature vectors and m denotes the lengths (L2 norm) of the vectors in Eq. 1 [44].

Let us demonstrate the computation of LINGO based TF cosine similarity using the sample SMILES strings *SMI*_1_ and *SMI*_2_, whose term frequencies are shown in Table 2. Since we have nine unique LINGOs, the lengths of the feature vectors are equal to nine, *m*=9. Each dimension in the feature vector represents the term frequency weight of the corresponding LINGO in the SMILES strings. Thus, the feature vectors for *SMI*_1_ and *SMI*_2_ are [1, 1, 1, 1, 0, 0, 0, 0, 0 ] and [0, 1, 1, 0, 1.3, 1, 1, 1, 1], respectively for the following order of the LINGOs [OC(O, C(O), (O) =, O) =O, CCCC, CCC(, CC(O, O) =C,) =C0]. Finally, the cosine similarity is computed as follows:

$${} {\fontsize{8.6pt}{9.6pt}\begin{aligned} Cos&Sim(SMI_{1}, SMI_{2})\\ &= \frac { \sum_{i=1}^{9} V_{SMI_{1},i} V_{SMI_{2},i}} {\lVert V_{SMI_{1}}\rVert \lVert V_{SMI_{2}}\rVert} \\ &= \frac { (1 \times 0) \,+\, \sum_{i=2}^{3} (1 \times 1) +\! (1 \times 0) + (0 \times 1.3) +\! \sum_{i=6}^{9} (0 \times 1) } { \sqrt[2]{ 4 \times \left(1^{2}\right)} \sqrt[2]{ 6 \times \left(1^{2}\right) + 1.3^{2}} } \\ &= 2 / 5.54 = 0.36 \end{aligned}} $$

#### LINGO based TF-IDF cosine similarity

Term Frequency-Inverse Document Frequency (TF-IDF) cosine similarity is the second model that we adopt to measure compound similarity by utilizing SMILES text. This method combines LINGO representation with the TF-IDF weighting-scheme. TF-IDF has originally been developed in the Information Retrieval domain for weighting the words in the documents just as TF weighting. This method is especially useful for filtering or assigning low weights to stop words such as ‘the’, ‘a’, and ‘an’. Words are the terms of a document corpus and each document is treated as a collection of terms (words). TF assigns higher weights to those terms that occur frequently in a document, IDF on the other hand, assigns higher weights to the rare terms in the document collection. Terms that are very common in the document collection are assumed to have little discriminating power. IDF is described as, $idf(t,D) = log \frac {N} {\left | { d \in D : t \in d} \right |}$ where *t*, *D* and *N* denote the term, document corpus, and number of documents in the corpus, respectively [45]. TF-IDF weighting is equal to the product of term frequency and inverse document frequency.

As shown in Eq. 1, the similarity between the feature vectors is computed by using cosine similarity. Each feature now contains the TF-IDF score of the corresponding term in the string. In this model, we treat each SMILES string as a document that comprises a set of LINGOs, and LINGOs are the terms of our system. LINGO length *q* is selected as four as it is in the original algorithm.

Let us illustrate this model by using samples from the compounds of the enzyme data set, which is one of the benchmark data sets used in this study [5]. As shown in Table 1, the enzyme data set comprises 445 different compounds each represented as unique SMILES strings. There are 1707 unique LINGOs created from 445 different SMILES strings. In other words, it is a system of 445 documents and 1707 terms.

For instance, “O)CO” and “(=O)” are two LINGOs. “(=O)” is a very frequent LINGO appearing in 300 out of the 445 SMILES strings. Its IDF is 0.17 and therefore, this LINGO can be considered as a stop word. “O)CO”, on the other hand, is a rather rare LINGO, which is included in only 18 SMILES strings. The IDF of this LINGO is 1.39.

The IDF weighting-scheme allows the model to assign importance to the rare LINGOs. SMILES strings that share infrequent LINGOs are favored and selected as more similar in this model. After term frequencies and IDFs of all the LINGOs are calculated, cosine similarity is computed to measure the similarity between two compounds.

Let us demonstrate the calculation of TF-IDF based cosine similarity by using our sample SMILES strings *SMI*_1_ and *SMI*_2_. The TF weights of the LINGOS in each string are computed as described in the previous sub-section (Section ‘LINGO based TF cosine similarity’). The IDF scores of the LINGOs, which are computed by assuming that the imaginary SMILES strings *SMI*_1_ and *SMI*_2_ are compounds in the enzyme data set, are shown in Table 3. Since the enzyme data set contains 445 compounds, the numerator in the IDF formula is 445. For a LINGO L, the denominator in the IDF formula is the number of compounds in the enzyme data set that contain the LINGO L. Thus, the TF-IDF weighted feature vectors for *SMI*_1_ and *SMI*_2_ are [ 2.3, 0.5, 0.6, 0.4, 0, 0, 0, 0, 0 ] and [0, 0.5, 0.6, 0, 1.04, 0.9, 1, 2, 1.9], respectively. The cosine similarity between them is computed as follows.

$${} {\fontsize{8pt}{9.6pt}\begin{aligned} &CosSim(SMI_{1}, SMI_{2}) \\ &= \frac { \sum_{i=1}^{9} V_{SMI_{1},i} V_{SMI_{2},i}} {\lVert V_{SMI_{1}}\rVert \lVert V_{SMI_{2}}\rVert} \\ &= \frac {0 + (0.5 \times 0.5) + (0.6 \times 0.6) + 0 + 0 + 0 + 0 + 0 + 0} { \sqrt[2]{2.3^{2}\,+\,0.5^{2}\,+\,0.6^{2}\,+\,0.4^{2}} \sqrt[2]{\!10.5^{2}\,+\,0.6^{2}\,+\,1.04^{2}\,+\,0.9^{2}\,+\,1^{2}\,+\,2^{2}\,+\,1.9^{2} }} \\ &= 0.61 / 8.2 = 0.07 \end{aligned}} $$

**Table 3:** The IDF scores for the LINGOs in the sample imaginary SMILES strings *S* *M* *I*_1_ and *S* *M* *I*_2_. The IDF scores are computed by assuming that *S* *M* *I*_1_ and *S* *M* *I*_2_ are compounds in the enzyme data set consisting of 445 compounds in total

LINGO Dictionary	IDF (log10(N/df))
OC(O	log10(445/2)
C(O)	log10(445/113)
(O) =	log10(445/105)
O) =O	log10(445/143)
CCCC	log10(445/61)
CCC(	log10(445/49)
CC(O	log10(445/36)
O) =C	log10(445/4)
) =C0	log10(445/5)

#### Composite kernels

Composite kernels are created by combining the similarity matrices obtained from two different methods, namely the 2D-based similarity function SIMCOMP and one of our SMILES-based similarity functions. *S*_*composite*_, representing the similarity matrix of the composite kernel, is derived by taking the unweighted average (i.e., *λ*=0.5) of the similarity matrix produced by SIMCOMP, *S*_*simcomp*_, and the similarity matrix *S*_*f*_ produced by a SMILES-based similarity function *f*,

$$S_{composite} = \lambda * S_{simcomp} + (1- \lambda) * S_{f} $$

To obtain *S*_*f*_, we experimented with our best performing SMILES-based similarity functions LINGO-based TF-IDF cosine similarity and LINGOsim (*q*=4).

### Experiment setup

Experiments followed the procedure proposed by Laarhoven [17]. For each interaction data set, five-fold cross validation is performed on the drug compounds. In other words, the data sets are divided into five equal-sized subsets and for each fold, one subset is used for testing while the system is trained with the remaining four subsets. This process is repeated five times.

## Results and discussion

In this study, 13 different similarity kernels that utilize SMILES (1D) representation of molecules are evaluated. In order to assess the performances of these drug kernels for the drug-target interaction prediction task, the WNN-GIP approach is adopted. We compare the results obtained using the SMILES representations of the compounds with the 2D-based similarity method, SIMCOMP, as well as two composite kernels formed by SIMCOMP and a 1D-based similarity method. In the original application of the WNN-GIP method, a compound similarity matrix was computed by the 2D representation based similarity method, SIMCOMP [17]. In this work, the similarity matrix is computed by SIMCOMP or the SMILES-based similarity functions. The compound similarity matrix is then processed by WNN-GIP to predict the protein-drug pairs in four different data sets. The enzyme data is the largest data set with more than 400 drugs and 600 proteins, while the ion channel and GPCR data sets are about half the size in terms of the number of drugs, and the nuclear receptor data set is the smallest with 54 drugs and 26 proteins.

A summary of the results for five-fold cross validation experiments for enzyme, ion channels, GPCR, and nuclear receptors data sets are presented in Table 4. The performances of the methods are compared using the Area Under the ROC Curve (AUC-ROC) and Area Under the Precision-Recall curve (AUC-PR) metrics. AUC-ROC presents the relation of True-Positive rate to the False-Positive rate, whereas AUC-PR shows the proportion of precision to recall. AUC-PR is favored when dealing with unbalanced data sets with one class domination [46]. Protein-drug interaction data sets contain small number of interacting pairs making them skewed.The AUC-ROC and AUC-PR results, with standard deviations given in parentheses, and the total time it takes to create a compound similarity matrix for each kernel are tabulated. The *p*-values for the statistical significance test comparing each method and SIMCOMP are given in Additional file 1: Table S1.

**Table 4:** Average AUC-ROC and AUC-PR scores for 5 repetitions of 5 fold cv. on each of the four data sets. The standard deviations are given in parenthesis

Method	AUC-ROC (std)	AUC-PR (std)	Time (sec)
**Enzyme**			
SIMCOMP	**0.863** (0.016)	0.303 (0.027)	413,7 min
Edit	0.833 (0.016)	0.178 (0.004)	6
NLCS	0.837 (0.014)	0.228 (0.013)	4
CLCS	0.834 (0.013)	0.234 (0.019)	331
SMILES-based substring	0.752 (0.006)	0.169 (0.010)	133
SMIfp CBD (34D)	0.846 (0.009)	0.199 (0.008)	1
SMIfp Tanimoto (34D)	0.832 (0.012)	0.191 (0.012)	1
SMIfp CBD (38D)	0.852 (0.009)	0.205 (0.009)	1
SMIfp Tanimoto (38D)	0.844 (0.012)	0.201 (0.006)	1
LINGOsim (q =3)	0.846 (0.013)	0.290 (0.013)	3
LINGOsim (q =4)	0.823 (0.010)	0.294 (0.006)	3
LINGOsim (q =5)	0.819 (0.015)	0.264 (0.013)	3
LINGO-based TF	0.811 (0.017)	0.259 (0.008)	19
LINGO-based TF-IDF	0.822 (0.012)	0.292 (0.031)	47
TF-IDF+SIMCOMP	0.852 (0.010)	**0.348** (0.017) ^*a*^	
LINGOsim+SIMCOMP	0.852 (0.016)	0.318 (0.019) ^*a*^	
**Ion Channels**			
SIMCOMP	**0.776** (0.012)	0.224(0.032)	48,7 min
Edit	0.754 (0.013)	0.199 (0.025)	1
NLCS	0.753 (0.007)	0.189 (0.037)	0,9
CLCS	0.755 (0.018)	0.185 (0.028)	47
SMILES-based substring	0.743 (0.004)	0.197 (0.031)	21
SMIfp CBD (34D)	0.717 (0.019)	0.136 (0.036)	0,3
SMIfp Tanimoto (34D)	0.698 (0.015)	0.125 (0.022)	0,3
SMIfp CBD (38D)	0.722 (0.012)	0.137 (0.024)	0,3
SMIfp Tanimoto (38D)	0.699 (0.028)	0.156 (0.028)	0,4
LINGOsim (q =3)	0.737 (0.015)	0.192 (0.046)	0,8
LINGOsim (q =4)	0.737 (0.011)	0.197 (0.037)	1
LINGOsim (q =5)	0.727 (0.009)	0.188 (0.026)	1
LINGO-based TF	0.738 (0.018)	0.204 (0.024)	3
LINGO-based TF-IDF	0.712 (0.014)	0.178 (0.029)	7
TF-IDF+SIMCOMP	0.763 (0.010)	**0.234** (0.017)	
LINGOsim+SIMCOMP	0.773 (0.012)	0.229 (0.018)	
**GPCR**			
SIMCOMP	0.867 (0.009)	0.307 (0.018)	71,2 min
Edit	0.844 (0.015)	0.248 (0.030)	1
NLCS	0.853 (0.006)	0.247 (0.013)	1
CLCS	0.855 (0.014)	0.279 (0.030)	52
SMILES-based substring	0.782 (0.019)	0.205 (0.032)	21
SMIfp CBD (34D)	0.852 (0.014)	0.209 (0.018)	0,3
SMIfp Tanimoto (34D)	0.847 (0.006)	0.213 (0.016)	0,3
SMIfp Tanimoto (38D)	0.856 (0.009)	0.228 (0.015)	0,3
LINGOsim (q =3)	0.875 (0.003)	0.317 (0.015)	1
LINGOsim (q =4)	0.876 (0.004)	0.333 (0.020) ^*a*^	1
LINGOsim (q =5)	0.874 (0.006) ^*a*^	0.337 (0.019) ^*a*^	1
LINGO-based TF	0.872 (0.004)	0.335 (0.012) ^*a*^	3
LINGO-based TF-IDF	0.871 (0.007)	0.348 (0.018) ^*a*^	9
TF-IDF+SIMCOMP	**0.885** (0.006) ^*a*^	**0.371** (0.017) ^*a*^	
LINGOsim+SIMCOMP	0.879 (0.009) ^*a*^	0.335 (0.016) ^*a*^	
**Nuclear Receptors**			
SIMCOMP	0.856 (0.015)	0.435 (0.008)	2,9 min
Edit	0.828 (0.009)	0.305 (0.029)	0,2
NLCS	0.815 (0.018)	0.302 (0.032)	0,2
CLCS	0.813 (0.037)	0.319 (0.039)	10
SMILES-based substring	0.766 (0.028)	0.335 (0.035)	2
SMIfp CBD (34D)	0.809 (0.026)	0.296 (0.015)	0,1
SMIfp Tanimoto (34D)	0.784 (0.031)	0.281 (0.020)	0,1
SMIfp CBD (38D)	0.815 (0.017)	0.307 (0.024)	0,1
SMIfp Tanimoto (38D)	0.787 (0.030)	0.322 (0.034)	0,1
LINGOsim (q =3)	0.800 (0.013)	0.351 (0.036)	0,2
LINGOsim (q =4)	0.829 (0.013)	0.414 (0.031)	0,2
LINGOsim (q =5)	0.834 (0.013)	0.389 (0.023)	0,2
LINGO-based TF	0.820 (0.013)	0.373 (0.035)	0,4
LINGO-based TF-IDF	0.855 (0.022)	0.418 (0.016)	0,8
TF-IDF+SIMCOMP	**0.861** (0.008)	**0.436** (0.049)	
LINGOsim+SIMCOMP	0.840 (0.015)	0.399 (0.031)	

The best AUC-ROC and AUC-PR results for each data set are indicated in bold. The results that are significantly better than SIMCOMP according to the paired t-test (*α* = 0.05) are indicated with ^*a*^. The *p*-values range between 0.0004 and 0.0329, and they are provided in the Additional file 1: Table S1.

An overall comparison of the AUC-ROC values shows that the 2D-based method SIMCOMP gives the best results for the enzyme and ion channels data sets. The edit distance performs as well as the other SMILES-based methods, even though it is one of the most basic approaches to measure similarity between two strings. The SMILES-based substring kernel performs significantly worse than the other kernels on the enzyme and GPCR data sets. A detailed investigation of the results obtained with each similarity method is presented below.

The NLCS and CLCS kernels perform close to the edit distance. The CLCS, which combines three different modifications of the NLCS function, does not significantly improve upon the original NLCS method for this domain. CLCS achieves significantly better AUC-PR results than NLCS only on the GPCR data set (*p*-value 0.019).

The original SMILES-based fingerprint approach (SMIfp) converts SMILES representation into a 34D fingerprint, where each dimension represents the frequency of a pre-determined character for a given SMILES string. In our study, we compared the frequent characters of our compound data sets with the original 34 character list of SMIfp. The comparison revealed that the characters ‘@’, ‘ ∖’, ‘/’, ‘.’ which are ignored in the original SMIfp approach, were among the most frequent characters in our data sets. In addition, the ‘%’ character, which is listed as frequent, was a rare character in our data set. ‘@’ and ‘@@’ characters give information on the chirality while ‘/’ and ‘ ∖’ are the directional bonds. Therefore, we decided to update the SMIfp design according to this information by adding five more characters, ‘@’, ‘@@’, ‘.’, ‘ ∖’, and ‘/’, and removing ‘%’. As a result, we created a 38D SMIfp, which was also tested with CBD and Tanimoto similarity coefficient. Both AUC-ROC and AUC-PR results show that 38D SMIfp performs significantly better than 34D SMIfp on the enzyme data set (*p*-values 0.0007 and 0.0016, respectively) when used with CBD and on the GPCR data set when used with Tanimoto (*p*-values 0.014 and 0.034, respectively). In addition, for 34D SMIfp, use of CBD provides a statistically significant advantage over Tanimoto on the enzyme and ion channels data sets in terms of AUC-ROC score with *p*-values 0.014 and 0.032, respectively.

LINGOsim (*q*=4) produces significantly better AUC-PR results than SIMCOMP with *p*-value 0.0017 on the GPCR data set (Table 4). AUC-ROC results show that LINGOsim with three character LINGO setting performs better than with four and five character settings on the enzyme data set, whereas setting substring length to five works better on the nuclear receptors data set, suggesting that the performance of LINGOsim with different substring lengths depends on the data set. Therefore, we may infer that it is better to test and see the best suitable setting for each data set.

Both the SMILES-based substring kernel and the LINGOsim kernels are based on partitioning the SMILES string into substrings to calculate similarity. While the LINGOsim method uses fixed length substrings, the other uses all possible lengths of substrings starting with two characters. The results show that use of different substring lengths for LINGOsim does not drastically change the results. Therefore, the success of LINGOsim over the SMILES-based substring kernel may be due to the fact that the SMILES-based substring kernel does not perform length normalization.

LINGO-based TF-IDF cosine similarity kernel produces significantly better AUC-PR results than SIMCOMP on the GPCR data set with *p*-value 0.009. This approach uses TF-IDF weighting and combines it with the LINGO representation of SMILES. It treats each SMILES string as a document and each LINGO in the SMILES is considered as a term. TF weighting produces comparable AUC-ROC and AUC-PR results with TF-IDF weighting for the GPCR and enzyme data sets. In the Nuclear Receptors data set, the TF-IDF based method performs significantly better (*p*-value 0.033) than the TF based method in terms of AUC-ROC scores, whereas in the Ion Channels data set the TF-based method produces a significantly better (*p*-value 0.030) AUC-ROC score than TF-IDF based method. The better performance of TF-IDF weighting over TF weighting suggests that, Nuclear Receptors data set might contain some distinguishable LINGOs.

The application of the TF-IDF weighting also allows us to further investigate the LINGO structures of each compound data set based on their IDF values. LINGOs with lower IDF values indicate that they are very common in the SMILES strings for the given data sets, similar to the stop words of a document corpus. Therefore, we list and investigate the top ten LINGOs with the lowest IDF values for each compound data set and provide the number of compounds they appear in Table 5. For instance, “(=O)”, carboxyl group, is a common LINGO in all data sets.

**Table 5:** Top 10 most common LINGOs of each compound data set

**Enzyme/445**		**GPCR/223**	
LINGO	Num. of drugs	LINGO	Num. of drugs
c0cc	321	c0cc	180
(=O)	300	0ccc	170
0ccc	279	(=O)	117
C(=O	228	cccc	108
ccc0	197	ccc0	107
cccc	171	ccc(	94
)c0c	155	C(=O	87
@H](	149	)c0c	84
ccc(	144	Cc0c	78
[C@H	144	C(O)	72
**Ion Channels/210**		**Nuclear Receptors/54**	
LINGO	Num. of drugs	LINGO	Num. of drugs
c0cc	165	(=O)	37
0ccc	148	[C@H	35
(=O)	130	C@H]	35
ccc0	116	C(=O	35
cccc	105	H]0C	35
C(=O	101	[C@@	35
)c0c	94	C@@H	35
ccc(	72	@@H]	35
O)c0	56	@H]0	35
=O)c	54	)[C@	34

As shown in Table 5 more than half of the top-ten LINGOs in the GPCR, enzyme, and ion channels data sets are shared. On the other hand, the top-ten LINGOs in the nuclear receptors data set are slightly different from these. We observe that substrings of “[C@@H]” are common LINGOs in the nuclear receptors data set. These symbols indicate tetrahedral chirality in clockwise direction, for example, at the C *α* carbon of the peptide bond.

We also tested two composite kernels in which we combine SIMCOMP with TF-IDF based cosine similarity and LINGOsim (q =4). Combination of SIMCOMP with TF-IDF based cosine similarity kernel produces the best AUC-PR results on all data sets. It also has better AUC-ROC scores amongst all other kernels on the GPCR and nuclear receptors data sets.

Except for the ion channels data set, the SMILES-based similarity methods perform almost as well as SIMCOMP, a 2D-based method using graph representation to measure similarity. In terms of time complexity, all SMILES-based methods perform significantly better than SIMCOMP. For instance, on the GPCR data set, while it takes more than an hour to compute the pairwise similarities among the compounds using SIMCOMP, it only takes one second when the LINGO kernel is used. Furthermore, LINGO (q =4) manages to achieve a comparable AUC-PR score with SIMCOMP, even though it only uses SMILES to measure similarity.

The improvement of the TF-IDF/SIMCOMP composite kernel over SIMCOMP, especially on the GPCR data set with a statistical significance in terms of both AUC-ROC and AUC-PR values (*p*-values 0.002 and 0.0005, respectively), shows that the TF-IDF based cosine kernel might be useful while combining different types of chemical similarity kernels.

In order to illustrate the improvement of the composite kernel over SIMCOMP, let us investigate a case in which an interaction was not predicted when SIMCOMP was used, but was successfully predicted when the composite kernel of SIMCOMP and TF-IDF was used: The interaction of Adrenoceptor alpha 2A (hsa150) with Phentolamine mesilate (D00509) (in the GPCR data set). The SMILES string of Phentolamine mesilate contains some rare LINGOs, namely “NCCN” and “ =NCC” with IDF values 1.8 and 1.9, respectively. Considering the IDF value of 0.3 for the most frequent LINGO (“c0cc”) in the GPCR data set, it becomes more apparent that these rare LINGOs represent some specific features of a compound. Further investigation of other drugs that interact with Adrenoceptor alpha 2A shows that, Brimonidine tartrate (D02076), also has the same two rare LINGOs and the interaction with this drug is successfully predicted by the composite kernel, but not by SIMCOMP alone. Similarly, the interactions of Adrenoceptor alpha 2A with Clonidine (D00281) and Clonidine hydrochloride (D00604), which contain the “NCCN” LINGO are also predicted successfully by the composite kernel. Therefore, we can suggest that these LINGOs reveal a pattern among the drugs that bind to Adrenoceptor alpha 2A. The inclusion of the TF-IDF kernel to the composite kernel helps us discover hidden patterns by highlighting such rare LINGOs.

## Conclusion

This work aims to provide a comparison of the available chemical similarity measurement methods that utilize SMILES representation of molecules. The methods presented here can be used in any model that requires the computation of compound similarity. In this study, we evaluated these methods using one of the state-of-the-art approaches in the drug-target interaction prediction task, namely WNN-GIP [17]. This model makes use of both chemical similarity of compounds and sequence similarity of proteins and is used on four different drug-protein data sets proposed by Yamanishi and coworkers [5]. In total, 13 string similarity functions including two novel drug similarity methods adopted from the Information Retrieval domain, namely cosine kernel with TF and TF-IDF weighting are tested to assess their performances in predicting protein-drug interactions. We also test two composite kernels created from SIMCOMP and two 1D kernels, TF-IDF based cosine similarity and LINGOsim, respectively. The results are compared with those found using the 2D representation based method SIMCOMP.

With the WNN-GIP method, an adjacency matrix is built using the bipartite drug-target interaction network. This adjacency matrix and the compound chemical similarity (calculated using the similarity kernels) and protein sequence similarity (calculated using the Smith-Waterman algorithm) are combined to convert drugs and targets into feature vectors. The bipartite drug-target interaction network includes information on pairs that are known to interact, but lacks information to differentiate between inactives (known absence of interaction) and untested compounds (absence of knowledge on interaction) and the limitation expressed in Laarhoven et al.’s work is present in this work as well [17]. The adjacency matrix can be enriched by including information about the strengths of the interactions in the form of IC50 values to include inactives.

The comparison of the results using 1D-based similarity methods with those found using the commonly used 2D-based similarity kernel SIMCOMP shows that the 1D-based methods of molecular similarity comparison perform almost as well as the 2D-based methods in the protein-drug interaction task. However, when the run times obtained using the two approaches are compared, 2D representation based descriptors, which use graph algorithms to compare similarity amongst molecules, are computationally more complex than the 1D SMILES representation, which is a string of characters. The experiments indicate that SMILES-based kernels are significantly faster than the 2D-based SIMCOMP. Using SMILES string as a molecular similarity kernel is not only fast and straightforward, but also more flexible since any string similarity algorithm can be applied to this representation. Furthermore, 2D and 3D representation of a molecule can be derived from its SMILES string by applying an efficient reconstruction algorithm [26]. Our study shows that 1D SMILES representation based methods perform close to SIMCOMP with significant computational flexibility and time advantage.

In this work inspired by the Information Retrieval domain, we proposed the application of cosine similarity with TF and TF-IDF weighting as novel ligand similarity kernels. For the GPCR data set, AUC-PR results show that the LINGO-based TF-IDF cosine similarity kernel performs slightly better than SIMCOMP in the task of protein-drug interaction prediction and LINGOsim with *q*=4 has comparable AUC-ROC results. Furthermore, the composition of TF-IDF based cosine similarity kernel with SIMCOMP proves to be promising given its AUC-PR results are the best amongst all kernels. Use of LINGO based TF-IDF weighting also allows identification of differences in the distribution of LINGOs in the compound data sets. It is observed that nuclear receptor drugs differ from the other drugs in terms of common LINGOs.

## Availability of the supporting information

The source code for the SMILES-based similarity algorithms and compound data are provided in the repository and publicly available at: https://github.com/hkmztrk/SMILESbasedSimilarityKernels.

## Additional file

Additional file 1
**n.** Excel file containing the *p*-values of the paired t-test comparing the AUC-ROC and AUC-PR score of each method and SIMCOMP for the methods that perform better than SIMCOMP. (XLSX 11.5 kb)
